# Supplementary figures and images for: The novel bacteriocin romsacin from Staphylococcus haemolyticus inhibits Gram-positive WHO priority pathogens
Source: Microbiol Spectr. 2023 Oct 31;11(6):e00869-23. doi: 10.1128/spectrum.00869-23 (PMC10715183; doi:10.1128/spectrum.00869-23)

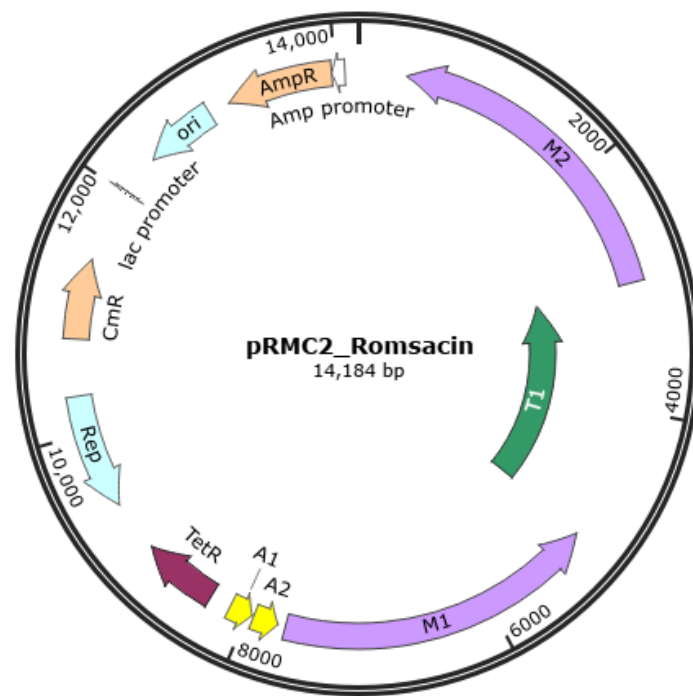

Supplementary Figure 1: plasmid pRMC2 (Addgene #68940).

Supplement: Supplementary Figure 1 — Plasmid pRMC2. [file spectrum.00869-23-s0001.pdf]
